# Biomimetic Materials Based on Poly-3-hydroxybutyrate and Chlorophyll Derivatives

**DOI:** 10.3390/polym16010101

**Published:** 2023-12-28

**Authors:** Polina M. Tyubaeva, Kristina G. Gasparyan, Roman R. Romanov, Evgeny A. Kolesnikov, Levon Y. Martirosyan, Ekaterina A. Larkina, Mikhail A. Tyubaev

**Affiliations:** 1 Department of Physical Chemistry of Synthetic and Natural Polymer Compositions, Emanuel Institute of Biochemical Physics, Russian Academy of Sciences, 4 Kosygina Street, 119334 Moscow, Russialevon-agro@mail.ru (L.Y.M.); 2 Academic Department of Innovational Materials and Technologies Chemistry, Plekhanov Russian University of Economics, 36 Stremyanny Per., 117997 Moscow, Russia; otmetkin@mail.ru (R.R.R.); tyubaeva.ma@rea.ru (M.A.T.); 3 Department of Chemistry and Technology of Biologically Active Compounds, Medicinal and Organic Chemistry, Institute of Fine Chemical Technology, MIREA-Russian Technological University, 119454 Moscow, Russia; 4 Department of Functional Nanosystems and High-Temperature Materials, National University of Science and Technology (MISIS), 119991 Moscow, Russia; kolesnikov.ea@misis.ru

**Keywords:** poly-3-hydroxybutyrate, chlorophyll derivatives, electrospinning, antibacterial properties, supramolecular structure

## Abstract

Electrospinning of biomimetic materials is of particular interest due to the possibility of producing flexible layers with highly developed surfaces from a wide range of polymers. Additionally, electrospinning is characterized by a high simplicity of implementation and the ability to modify the produced fibrous materials, which resemble structures found in living organisms. This study explores new electrospun materials based on polyhydroxyalkanoates, specifically poly-3-hydroxybutyrate, modified with chlorophyll derivatives. The research investigates the impact of chlorophyll derivatives on the morphology, supramolecular structure, and key properties of nonwoven materials. The obtained results are of interest for the development of new flexible materials with low concentrations of chlorophyll derivatives.

## 1. Introduction

Tetrapyrroles and their derivates are the basis of essential physiological functions in most living organisms [1]. In this context, tetrapyrroles and their derivatives are of a great interest for the creation of new materials and biomedical tools [2,3]. 

Scientists have achieved great success in the synthesis and modification of tetrapyrroles through the directional design of photosensitive molecules via varying the nature of the metal, peripheral substituents, or the length of the conjugated double bond system [4]. It is important to emphasize the successes in the organic synthesis of chlorophyll and its derivatives [5]. Significant attention is drawn to chlorophyll derivatives due to their set of unique properties.

Chlorophyll’s functions include accumulation, transport of energy, and driving charge separation reactions in reaction centers [6]. It possesses antioxidant, antigenotoxic, and antimutagenic properties [7,8,9]. Its derivatives are bioactive and easily modified, and their synthesis cost is low [5,10,11]. Industrial forms of chlorophyll are well known, and they are successfully used in the food industry, in cosmetics, and in medicine [9,12].

One particular success is the effective use of chlorophyll derivatives in photodynamic therapy [5]. Their effectiveness is due to combination of light, photosensitizer, and cytotoxic agent [13]. In case of chlorophyll derivatives, the cytotoxic agent is the molecular oxygen which only is produced upon in situ irradiation [14]. So, it is possible to effectively control the work of such remedies for therapeutic purposes.

Nanoparticles based on chlorophyll derivatives for photodynamic therapy are well known due to their good photosensitizing properties caused by the energy transfer from the nanoparticle to the photosensitizer [15].

Despite the tendency of porphyrins to accumulate in tumor cells during photodynamic therapy, the problem of effective delivery of porphyrins to targets remains unresolved. Due to the limitation of the radius of action of porphyrin molecules, it is important to accurately understand the localization of the photosensitizer, as well as to control its concentration [16]. In addition, it is important to control the hydrophilicity of the material containing the photosensitizer, since the photosensitizer molecules are highly hydrophobic, which makes it difficult for them to penetrate into the cell through the cell membrane [17]. 

The increased export of active drugs to the cancer cell leads to an increase in its resistance, depending on the form of cancer [18]. Therefore, the problem of creating flexible, non-toxic, and biocompatible carriers capable of delivering small concentrations of dosage forms to the target has not been solved. Even well-known solutions cannot be delivered to the target cell with the correct dosage without high toxicity [19,20]. In addition, the need remains for more flexible carriers for an easy and fast development of delivery systems with the appropriate design.

One of the effective solutions to this problem is the creation of various delivery systems based on biopolymers with a variable degradation period and high biocompatibility.

In general, success in combining porphyrins and polymers as carriers has been observed in a large number of scientific papers. Systems with polymers such as chitosan [21], polyethylene glycol [22], and poly(lactide-co-glycolide) [23,24] are well studied. But special attention should be paid to a polymer such as poly-3-hydroxybutyrate (PHB) [25,26]. PHB has many advantages over other polymers, among which the key ones are high biocompatibility, stability of properties, and controlled biodegradation in a wide temperature range [27,28].

Such materials have found wide application in photodynamic cancer therapy. However, problems remain unresolved, both in cancer therapy and in other areas of biomedicine. Among the unresolved problems in cancer therapy, of note are potential toxicity, the weak binding of the therapeutic agent with the carrier, and the difficulty of modifying carriers [29]. The search for a solution has led many scientists to biomimetic structures in view of the possibility of obtaining unique nature-like and easily modifiable carrier materials for each specific case [30].

Biomimetics are materials which imitate structures of natural origin to improve human lives [31]. In comparison with classical nanomaterials, biomimetics are able to present higher biocompatibility and biodegradability, enhanced targeting efficacy, and, due to their unique structure, side effects on normal cells can be reduced [32,33].

In this work, we decided to create biomimetic materials based on PHB and various chlorophyll derivatives for a detailed study of their suitability for the production of polymer–porphyrin systems. As a method of obtaining biomimetic systems, electrospinning (ES) was chosen. ES allows us to achieve maximum similarity of the created materials to the structures produced by nature due to the formation of a highly developed hierarchical structure of fibrous material [34]. Biomimetic materials based on PHB–porphyrin electrospun composites are already known: polyhydroxybutyrate/5,10,15,20-tetrakis(4-hydroxy-phenyl)-21H,23H-porphine [35], polystyrene/polyhydroxybutyrate/graphene/tetraphenylporphyrin [36], polyhydroxybutyrate/Hemin [37], and polyhydroxybutyrate/tetraphenylporphyrin [38]. So, in different PHB/porphyrin combinations, the resulting materials are biocompatible, biodegradable, and could be easily modified. Moreover, ES of PHB/porphyrin systems may allow us to obtain flexible materials, which is very important for preparing effective carriers.

Thus, the aim of the work was to establish the effect of chlorophyll derivatives on the PHB matrix of electrospun biomimetic systems.

It is especially interesting whether there is a prospect of using such PHB/chlorophyll-derivative electrospun materials in biomedicine. For instance, one of the proposed applications could be the delivery of minimum porphyrin concentrations to the target zones. Clinical studies should be preceded by a comprehensive study of the properties of the polymer, as well as the polymer–porphyrin system, so there may be cases of low stability of the polymer matrix when various concentrations of photosensitizer are introduced into it. Morphological, mechanical, chemical, and thermophysical properties were selected in order to analyze the most important properties of the polymer matrix. In addition, the hydrophobicity of materials, as well as antimicrobial properties in the absence of excitation, are considered in the work. This characterization of the properties will allow us to choose the best polymer–porphyrin combinations, as well as consider the possibilities of varying the concentrations of photosensitizers from lower to higher and maximum.

## 2. Materials and Methods

### 2.1. Materials

**Figure 1:**
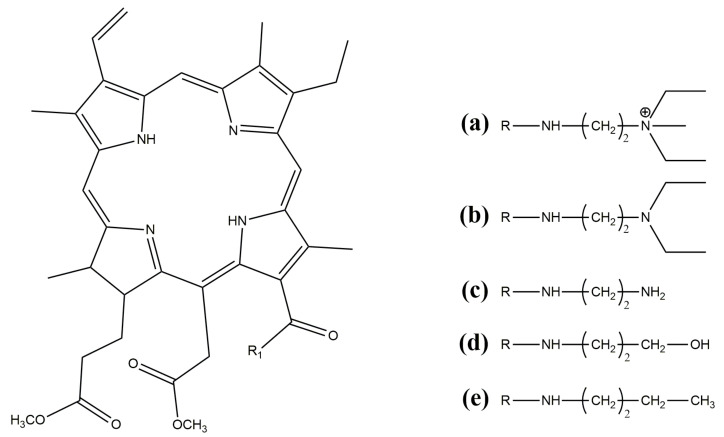
Structural formulas of chlorophyll derivatives: (**a**) mC_2_N+; (**b**) mC_2_N; (**c**) mC_2_NH_2_; (**d**) mC_3_OH; (**e**) mC_4_.

For this study, we chose biopolymer poly-3-hydroxybutyrate (PHB), obtained through microbiological synthesis (16F series, produced by BIOMER, Frankfurt, Germany). This polymer exhibited a crystallinity of 60%, a molecular weight of 206 kDa, a density of 1.248 g/cm^3^, and a melt flow index of 10 g/10 min (180 °C, 5 kg). Powdered derivatives of chlorophyll, namely mC_2_N+, mC_2_N, mC_2_NH_2_, mC_3_OH, and mC_4_ (Figure 1), were utilized as functional additives. The synthesis of the amide derivatives of chlorin e_6_ from pheophorbide, a methyl ester, was described in detail earlier in articles: [39] for compound mC_3_OH, [40] for compound mC_4_, and [41] for compound mC_2_NH2. 13^1^-N-(2-N’, N’-diethylaminoethyl) amide-15^2^,17^3^-dimethyl ester of chlorin e_6_ (mC2N(Et)2) was synthesized and then quaternized with methyl iodide (mC_2_N(Et)_2_Me^+^ I^−^) according to the reported procedures in [42]. Both substances were dissolved in chloroform (Biolot, St. Petersburg, Russia) for the experiments.

### 2.2. Methods

#### 2.2.1. Obtaining of Nonwoven Fibrous Materials

**Figure 2:**
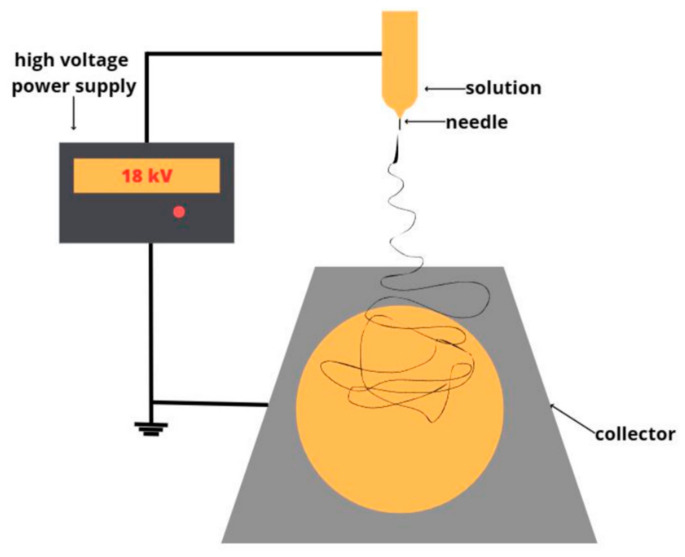
Single-capillary ES setup.

Fibrous nonwoven materials were obtained using the electrospinning (ES) method utilizing a single-capillary setup (Figure 2). Homogeneous solutions were prepared, including a 7% concentration solution of PHB and chlorophyll derivatives dissolved in chloroform with an additive concentration of 0.03% (the lowest concentration possible for preparation without limiting miscibility and controlling the uniformity of the additive distribution in the solution). Each solution was used in a volume of 25 mL.

The forming process proceeded as follows: under the influence of electrostatic force, fibers were drawn from the solution in the cell and deposited on a substrate located on the lower electrode. During the forming process, the solvent evaporated and the fiber solidified. The obtained nonwoven materials were subjected to drying to remove solvent residues and excess moisture for 48 h at 24 °C. The gas pressure on the solution was 10 kg(f)/cm^2^. The air was used as the gas. The diameter of the capillary was 0.1 mm. ES was carried out in an isolated chamber at a relative humidity of 40%, a temperature of 24 °C, and an air velocity less than 0.2 m/s. The distance between the electrodes was 210 mm for PHB and 220 mm for PHB/chlorophyll derivatives and was selected experimentally.

Several methods were chosen for investigating biomimetic materials. Morphology and structure were investigated using scanning electron microscopy. The effect of additives on the course of electrospinning was assessed not only visually but also with the most important operational properties for nonwovens, namely permeability and strength. The most significant characteristics of the polymer, which include the chemical structure, as well as its stability after the introduction of photosensitizers and the supramolecular structure, which often undergo significant changes with the introduction of additives, were studied using Fourier-transform infrared spectroscopy and differential scanning calorimetry. Antimicrobial properties are good indicator of the activeness of photosensitizers in the material, so they were also studied.

#### 2.2.2. Scanning Electron Microscopy (SEM)

Scanning electron microscopy (SEM) was chosen to investigate the morphology of obtained materials. Images of electrospun PHB/chlorophyll derivatives composites were obtained using the Tescan VEGA SBU II (Brno, Czech Republic) on samples coated with a platinum layer for 10 min. Structural changes, including variations in fiber diameter, defects, and morphology, were analyzed using the Olympus BX43 microscope (Olympus, Tokyo, Japan). The primary morphological properties of the fibers were measured through micrography, as facilitated with Olympus Stream Basic software.

#### 2.2.3. Differential Scanning Calorimetry (DSC)

Differential scanning calorimetry (DSC) is an experimental method used to study the thermal properties of materials. This method was chosen to investigate structural changes in the crystalline phase of the polymer matrixes under influence of the additives. In this study, the DSC 214 Polyma (Netzsch, Selb, Germany) was employed. The methodology involved two heating cycles (from 20 °C to 220 °C) and two cooling cycles (from 220 °C to 20 °C) with a temperature change rate of 10 K/min; samples were analyzed in an argon atmosphere. DSC measures the heat flow absorbed or released by a substance as its temperature changes, so this method allows for the determination of heat capacity, thermal transitions, and crystalline and amorphous properties of the material.

Crystallinity degree, χ, was calculated from normalized peak enthalpies from the melting peak as follows:

(1) $$\chi =\frac{\Delta H}{H_{PHB}}\times 100\%$$

where Δ*H*—melting enthalpy; *H_PHB_*—melting enthalpy of the ideal crystal of the PHB, 146 J/g [43].

#### 2.2.4. Fourier-Transform Infrared Spectroscopy (FTIR) 

Fourier-transform infrared spectroscopy (FTIR) was chosen to investigate changes in the chemical structure of PHB and to understand if there is any specific chemical interaction between chlorophyll derivatives and PHB. The spectra of the samples were obtained using the Lumos BRUKER infrared spectrometer (Karlsruhe, Germany) employing the multiple attenuated total internal reflection method on a diamond crystal. The spectral resolution was set at 2 cm⁻¹. The analysis was conducted in the wavelength range from 400 to 4000 cm⁻¹. This method enabled a detailed examination of the material’s interaction with infrared radiation, providing valuable insights into its chemical structure and composition.

#### 2.2.5. Wettability 

Wettability is characteristics of hydrophobicity of the material which allows for an understanding of the operational properties of the material. The surface wettability of PHB/chlorophyll derivatives samples was measured using the contact angle (CA) method, which quantifies surface moisture. Water droplets (2 µL) were dispensed onto the material’s surface using an automatic dispenser, and the contact angle formed between the droplet and the surface was measured. We utilized an optical microscope, M9 №. 63649, with an FMA050 lens (AmScope, Moscow, Russia), and analyzed the data using Altami Studio v3.4 software. The measurement was carried out using five drops in three different areas on the surface of the nonwoven material.

#### 2.2.6. Air Permeability

Air permeability is an important characteristic for understanding the open pores volume, which plays a significant role for planning flexible morphology and controlled resorption of materials. The air permeability of membranes, represented by the Gurley number, was measured following the standard protocol [44]. The applied pressure was 1.22 MPa, the air volume was 100 mL, and the test area was 6.5 cm^2^. The relative experimental error was ±5%.

#### 2.2.7. Antimicrobial Properties

Antimicrobial activity of the samples was studied using the O157:H7 strain of *Escherichia coli*. The lysis zone was considered a marker of antimicrobial activity. The Petri dishes were inoculated with a standardized inoculum of test microorganisms. The samples were placed into the Petri dishes. The strain was grown in BSA/agar at 37 °C and allowed to stay with the samples for 24 h.

#### 2.2.8. Mechanical Properties

The mechanical properties of the polymeric material, such as strength (the maximum load the material can withstand) and elongation at break (how much the material can stretch before breaking), allow us to understand the operational properties of the material and give a representation of the polymer state in fibers. Mechanical properties were measured using the testing machine DVT GP UG 5. The stretching speed was 25 mm per minute. Elongation at break was calculated as the difference between the final and initial length of the sample, expressed as a percentage of the initial length. The results were averaged based on no fewer than 5–7 measurements, with a measurement error of ±0.2%.

## 3. Results

### 3.1. Morphology of PHB/Chlorophyll Derivatives Electrospun Materials

Table 1 shows the most important parameters of the ES process, which differs for forming solutions of PHB/chlorophyll derivatives. Introduction of the additive had an effect on the viscosity of the forming solution, increasing it by an average of 20% for all PHB/chlorophyll derivative combinations. Voltage changed slightly with the addition of chlorophyll derivatives. It increased by 5% in case of PHB/mC_2_N and decreased by 5% in all other cases. A similar trend of value growth was observed for the flow rate of the forming solutions during the ES process. Flow rate increased by 10% in case of PHB/mC_2_N. In other cases, flow rate was unchanged (PHB/mC_2_NH_2_ and PHB/mC_3_OH) or decreased by 3% in case of PHB/mC_4_ and by 27% in case of PHB/mC_2_N+.

**Figure 3:**
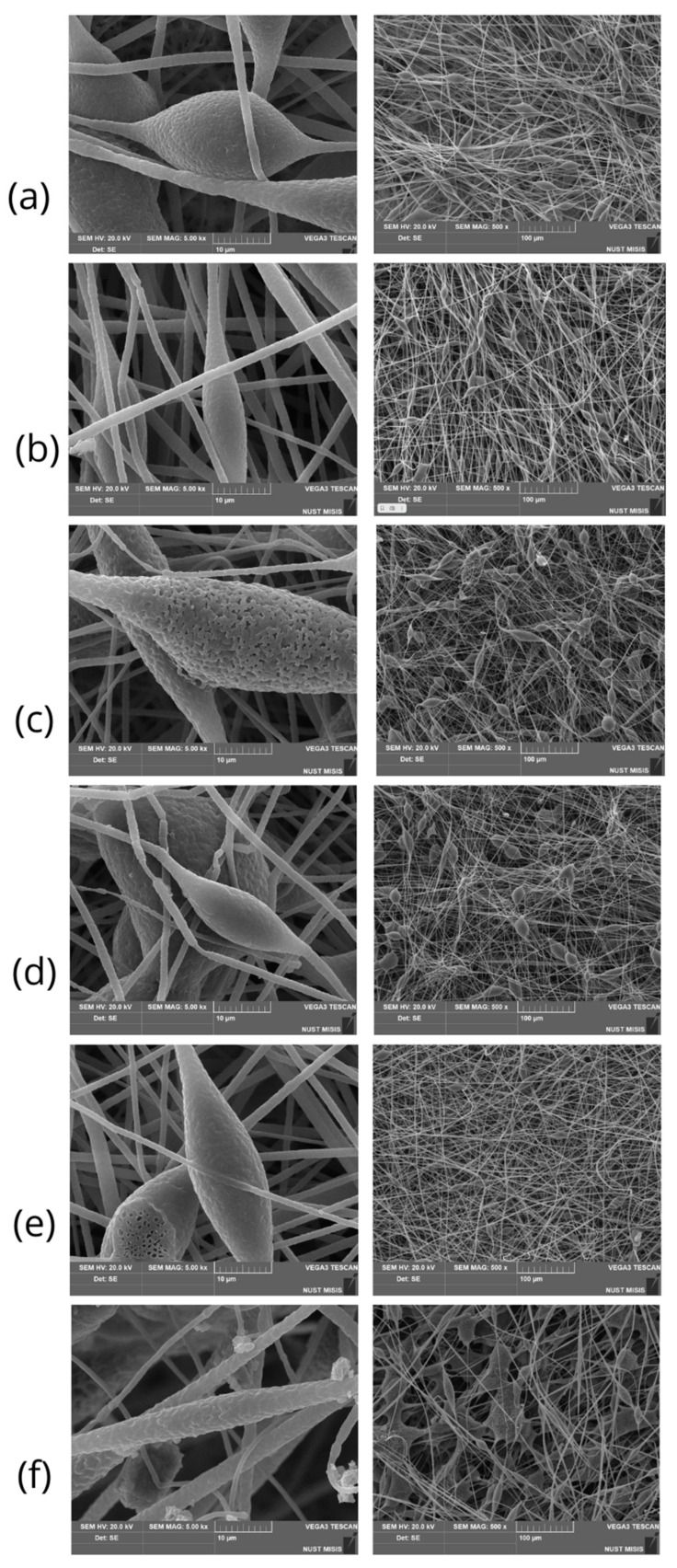
SEM images of electrospun materials based on PHB with different chlorophyll derivatives: (**a**) PHB; (**b**) PHB/mC_4_; (**c**) PHB/mC_3_OH; (**d**) PHB/mC_2_NH_2_; (**e**) PHB/mC_2_N+; (**f**) PHB/mC_2_N.

Figure 3 shows SEM images of electrospun materials based on PHB with different chlorophyll derivatives. A highly developed fibrous structure is clearly visible in all images. 

In the case of materials based on PHB/chlorophylls, it should be noted that PHB/mC_4_ (Figure 3b) and PHB/mC_2_N+ (Figure 3e) allowed us to minimize and almost completely reduce the number of defects, respectively. In these cases, the number of thickenings is minimal, there is no gluing, and the fibers are well cured. Meanwhile, other chlorophylls did not show a satisfactory result; moreover, they even worsened the quality of the material. It is important to note that in all cases, the Taylor cone and the ES process were stable, except PHP/mC_2_N.

Table 2 shows the most significant parameters of the morphology of the fibrous material. It is clearly seen that in all cases, the additives contributed to a decrease in average fiber diameters, and only mC_2_N had the opposite effect. In addition to the defects that made the material brittle and unusable, the diameter of the fibers increased by 1.4 times in the case of PHB/mC_2_N. For the rest of the additives, there was some variation in fiber distribution, but, in general, the average diameter of the fibers decreased by about 1.5 times. The smallest diameter was observed for the mC_3_OH sample with the highest porosity. All additives led to a decrease in bulk density of the material by about 1.5 times; however, there was no obvious correlation between the average diameter of the fibers and the density of their laying, as can be seen from the results (Table 2).

Changes in the porosity of the material and the proportion of open pores were estimated using the Gurley method. It can be clearly seen that the introduction of the additive significantly increased the proportion of open pores capable of passing a larger volume of air per unit of time (Table 2). PHB/mC_2_N was not able to withstand the experiment because of its high fragility. The air permeability of the electrospun materials increased by 6 times in case of PHB/mC_4_, by 7 times for PHB/mC_2_N+, by 9.8 times for PHB/mC_2_NH_2_, and by 11 times for PHB/mC_3_OH.

### 3.2. Chemical Structure PHB/Chlorophyll Derivatives Electrospun Materials

**Figure 4:**
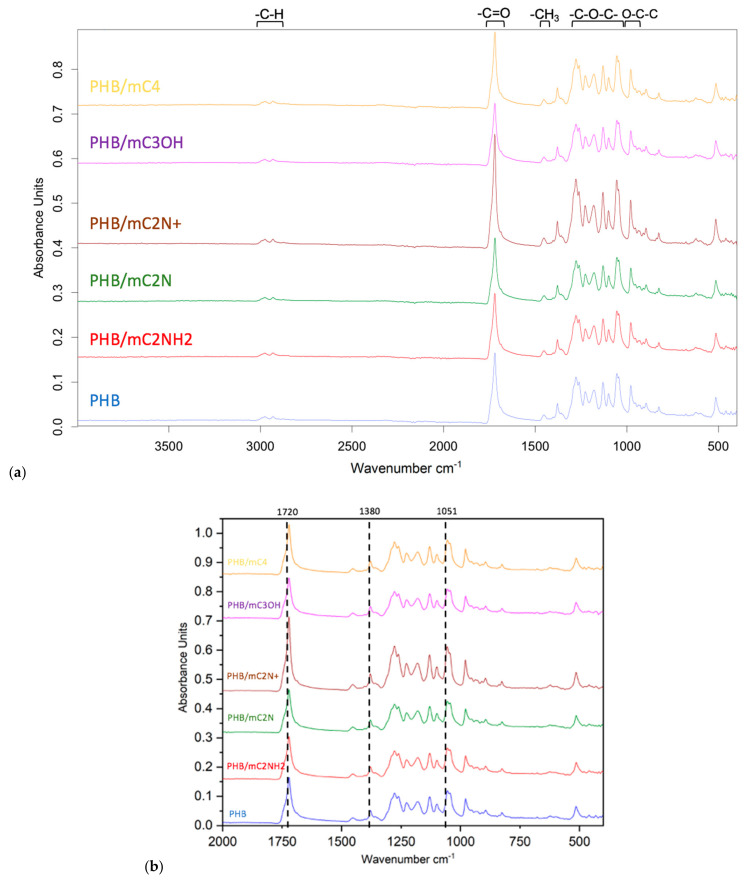
FTIR spectra of electrospun materials based on PHB with different chlorophyll derivatives: (**a**) the region 400–4000 cm^−1^, (**b**) the region 400–2000 cm^−1^.

Figure 4 shows FTIR spectra of PHB/chlorophyll derivatives electrospun materials. It can be clearly seen that the obtained spectra are identical to the spectrum of pure PHB. 

### 3.3. Thermophysical Characteristics of PHB/Chlorophyll Derivatives Electrospun Materials

**Figure 5:**
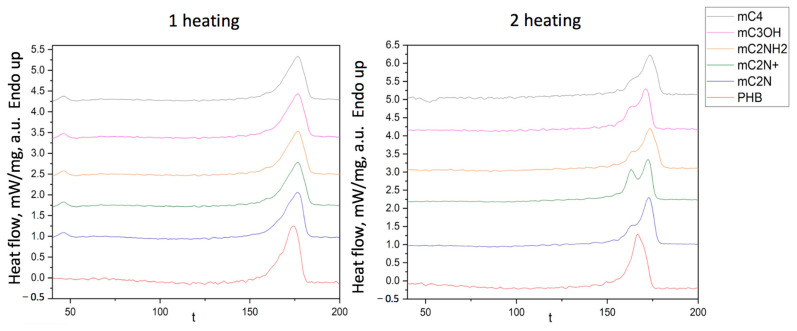
DSC curves of electrospun materials based on PHB with different chlorophyll derivatives.

DSC curves of PHB/chlorophyll derivatives electrospun materials are shown in Figure 5.

The enthalpy and melting temperature of PHB/chlorophyll derivatives electrospun materials are shown in Table 3. The temperature of melting of PHB had no impact in the case of PHB/mC_3_OH. In other cases, the melting point was slightly higher by 1–2%. But, at the same time, the enthalpy of melting noticeably decreased. PHB/mC_3_OH was characterized by the lowest enthalpy, 77.4, which was 17% lower than that for pure PHB. In other cases, the enthalpy was lower by 8–10%. A similar trend was detected for the second heating.

### 3.4. Wettability of PHB/Chlorophyll Derivatives Electrospun Materials

**Figure 6:**
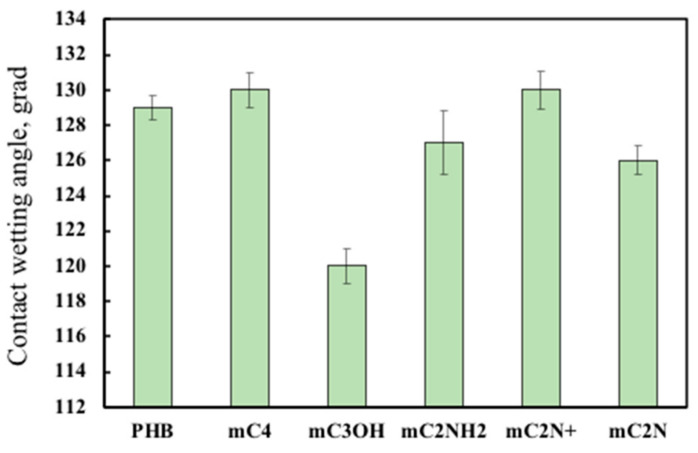
Wettability (contact angle) of electrospun materials based on PHB with different chlorophyll derivatives.

Figure 6 shows the changes in wettability of PHB-based electrospun materials with different chlorophyll derivatives.

As can be seen from Figure 6, chlorophyll additives affect the wettability of the material in different ways, leading to a change in its surface characteristics. The addition of mC_3_OH increases hydrophilicity by 8%, mC_2_NH_2_ by 3%, and mC_2_N by 4%. The addition of mC_2_N+ and mC_4_ decreases hydrophilicity by 1%.

### 3.5. Antimicrobial Properties of PHB/Chlorophyll Derivatives Electrospun Materials

**Figure 7:**
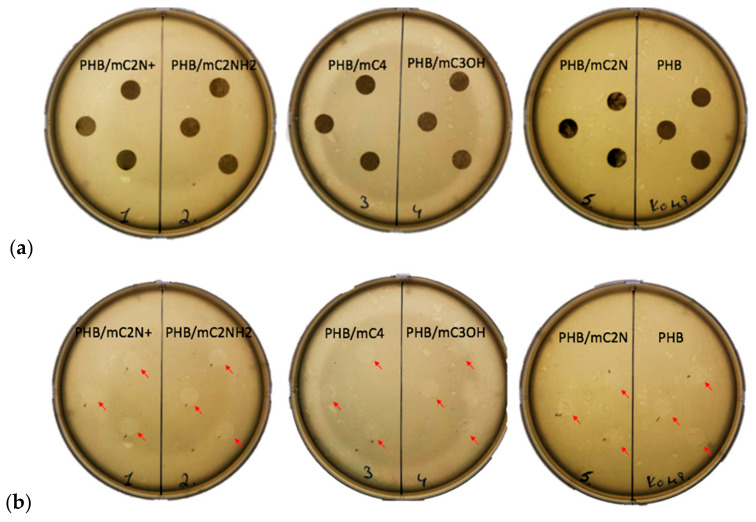
The microbiological test of electrospun materials based on PHB with different chlorophyll derivatives against *E. coli* (red arrows show lysis zone): (**a**) Petri dishes with PHB/chlorophyll derivatives samples (dark rounds), (**b**) Petri dishes without samples.

Antimicrobial properties of PHB/chlorophyll derivatives are shown in Figure 7.

### 3.6. Mechanical Properties of PHB/Chlorophyll Derivatives Electrospun Materials

Mechanical properties (tensile strength and elongation at break) are shown in Table 4. Unfortunately, only two samples—PHB/mC_4_ and PHB/mC_2_N+—were suitable for investigation on the test machine. Other samples (PHB/mC_3_OH, PHB/mC_2_NH_2_, and PHB/mC_2_N) were ripped apart without any signals.

**Figure 8:**
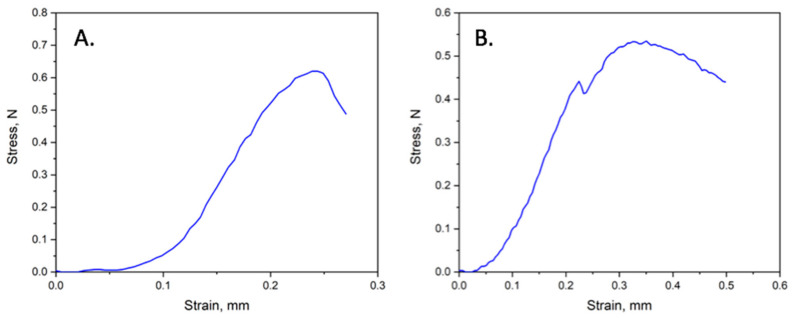
Mechanical analysis of PHB/chlorophyll derivatives: (**A**) PHB/mC_4_; (**B**) PHB/mC_2_N+.

Examples of stress–strain curves of PHB/mC_4_ and PHB/mC_2_N+ electrospun materials are shown in Figure 8. It can be seen that the mechanical properties are influenced by the strength of individual fibers, which are still brittle, although well cured (Figure 8B).

## 4. Discussion

In the work, electrospun materials with the lowest possible concentration (0.03%) of chlorophyll derivatives based on PHB were obtained. It is noteworthy that the utilization of a low additive concentration during the electroforming process has yielded significant benefits.

Different types of carrier tissues lead to skin photosensitivity, which causes damage to the organism [45]. There is a wide variety of nanocarrier platforms [46,47]. A lot of effort is aimed at obtaining multifunctional nanocarrier platforms [48]. Among the priority properties, changeable shape, controlled release, and hierarchical targeting should be especially mentioned [47]. These properties can be satisfied using carriers derived from biomimetic materials with regulated morphologies. The current study has shown that ES of PHB with low concentrations of chlorophyll derivatives will enable achieving a flexible, variable, and variant morphology, which is the first step in planning an effective carrier for small concentrations of active drugs.

In the current work, five chlorophyll derivatives (Figure 1) are considered, differing in the structure of the R1 radical. The types of R1 are shown in Figure 1. A number of studies have shown that additives containing polar groups, even in very small concentrations (proportion of percent), have a significant effect on forming properties [49,50]. The chlorophyll compounds considered in this work are distinguished by the presence of such polar groups. They contribute to improving the electrical conductivity of the solution, and, as a result, the ability to form fibers at a higher voltage with a higher efficiency. A higher voltage contributes to the formation of thinner fibers; however, it may be accompanied by an increase in the flow velocity of the solution jet, resulting in fibers with a thicker diameter [51]. In this regard, it is important to ensure optimal viscosity of the solution, as well as the pressure of the air column on the spinning solution. All of this underscores the importance of optimizing electroforming parameters, such as voltage, solution viscosity, and air pressure, to achieve a highly developed material structure.

It is important to note that despite the very small percentage of the additive (0.03%), a significant effect on the material was mentioned. In all five cases, the formation of a highly developed material structure was observed. However, differences should be noted. Figure 3 shows SEM images of PHB-based electrospun materials with different chlorophyll derivatives.

Nonwoven materials based on PHB are distinguished by the presence of specific defects, notably thickening (Figure 3a). Such defects are often found for pure PHB fibrous materials. In a number of works, scientists have used various methods of reducing thickenings: preparing PHB-based blends with other polymers, such as, for instance, PHB/PEG in works by Thanh et al. [52] or PHB/PCL in works by Borisova et al. [53]; using plasticizers like polyanilines in works by Ahmed et al. [54] or oligomeric lactic acid in works by Arrieta et al. [55]; using nanoparticles in works by Romero et al. [56] and others. 

Electrospun materials can be characterized as fibrous materials with highly developed surface areas. Such materials have a high degree of similarity to living structures, that is, they are biomimetic. The key to such a highly developed complex hierarchy of structure is morphology. The morphology of the fibers largely depends on the conditions of ES. The key parameters of ES include voltage, properties and flow rate of the spinning solution, the distance between the electrodes, and the pressure exerted on the solution [57]. Table 1 shows the most important parameters of the ES of the PHB/chlorophyll derivatives fibers. The capillary configuration has a great influence on the formation of fibers of the set diameter; it remained constant at 0.1 mm. Other important parameters were selected experimentally.

Solution viscosity significantly affects the droplet shape and the jet trajectory during the ES process. Increasing solution viscosity has been associated with the production of larger-diameter fibers [58]; however, despite the fact that all additives increased the viscosity of the system by 20%, the diameters changed randomly, which should be attributed to the difference in the chemical structure of the additives. Thus, the presence of polar groups can significantly affect the electrical conductivity of the solution, and an increase in electrical conductivity can lead to a decrease in diameters.

Flow rate has a great influence on morphology. Thus, the maximum flow rate of 7.2 led to the formation of film-like inclusions, and a decrease in the flow rate below 6.6 as in pure PGB made it possible to minimize defects in the form of thickenings. A noticeable effect on the flow rate of such low concentrations may be due to the structure of the R1 radical. Moreover, the difference in the influence of the R1 radical on the properties of the whole system of PHP/chlorophyll derivatives was observed in other important properties of the material.

In all cases, the PHB concentration of 7% wt. was used, which is the most optimal for a molecular weight of 460 kDa. The optimal concentration range for the ES of PHB lies in the range of 5–9% [51,59]. The selected concentration of 7%, which allowed us to achieve a balance in the viscosity of the forming solution, ensures an optimal flow rate of the solution. At the same time, lower concentrations have low forming properties, often forming smudges, and higher concentrations lead to the need to optimize the ES process. 

Of course, these changes significantly affected the morphology of the fibers (Figure 3). PHB/mC_2_N should be highlighted separately (Figure 3). The formation process of the PHB/mC_2_N solution was hindered; the fibers did not adhere properly to the substrate due to possible high levels of moisture on their surface, leading to the formation of glues and film-like formations (Figure 3f). In addition, PHB/mC_2_N had a critically high flow rate of 7.2 mL/min, which led to leakage of the solution and the formation of marked defects.

PHB/mC_2_N+ and PHB/mC_4_ had the lowest flow rate, which made it possible to obtain uniform fibers without defects. In general, it can be seen that the reduction in the flow rate of the polymer solution has a positive effect on the formation of fibers. It could be assumed that the lower flow rate leads to the reduce in the number of pear-shaped defects on the surface of the fibers.

PHB/mC_3_OH (Figure 3c) promotes the formation of highly porous fibers, which is usually associated either with a high rate of solvent evaporation [54] or with excessive friability of the amorphous phase due to the intermolecular interaction of the polymer and the additive [60]. Meanwhile, PHB/mC_2_N (Figure 3f) shows a significant increase in defects in the form of glues and smudges that make the material unusable, reducing its mechanical properties to a minimum.

As mentioned earlier, the Taylor cone and the ES process were stable during ES of all PHB/chlorophyll derivatives, except PHP/mC_2_N. This observation should be explained with an increased flow rate, which led to an increase in the voltage up to 19 kV, but at the same time, it did not allow us to avoid substructures such as smudges forming in the material layer due to instable ES. 

PHB electrospun materials are characterized by low air permeability (0.38 mL). This is due to the peculiarity of the forming of fibers and the formation of a large number of fiber-to-fiber glues, through which no air can freely pass. The introduction of chlorophyll derivatives significantly increased the breathability of the materials. This may be the result of structural changes in the polymer matrix caused by the introduction of even small concentrations of the additive.

This characteristic directly demonstrates the importance of the introduction of additives for the planning of high-pored materials. Thus, the highest values were shown by materials containing mCNH_2_ and mC_3_OH, where the smallest average diameters were detected. To our surprise, the materials with the least number of defects, PHB/mC_4_ and mC_2_N+, showed a lower increase in the proportion of open pores, which may be due to a greater degree of surface development and a smaller proportion of open space between the pores due to high fiber entanglement.

At the same time, in all cases except for mC_2_N, the material had a large proportion of open pores, which may indicate accelerated evaporation of the solvent during the formation of the fibers. Given that the solvent evaporates at the same rate in all systems, this should be attributed to the faster crystallization of PHB as a result of the presence of possible centers of crystallization in the form of porphyrin molecules.

Thus, the DSC method confirmed that the introduction of additives affected the nature of crystallization. All additives contributed to a decrease in the proportion of the crystalline phase to varying degrees. Moreover, it is important that part of the crystal fraction was poorly crystallized and formed a double peak of melting during the second heating. The type of radical had a distinctive impact on the structure of PHB, as evidenced by the appearance of a low-temperature shoulder during the second heating (155–165 °C), indicating the formation of defective crystallites or a fine crystalline fraction of fibers.

Additives probably occupy a position in the amorphous phase of the polymer, influencing the crystallization process and contributing to the formation of two fractions of PHB crystallites, large and small, after the first heating. However, with the slow forming during electrospinning, crystallization centers can act in all cases, except for mC2N, which already has a pronounced shoulder at the first heating. This effect is probably manifested in the formation of defects in the form of glues and smudges on the material (Figure 3f). And the material that differed in the smallest number of defects and the smallest bulk density (Figure 3e) has the most pronounced peak consumption, which indicates the significant role of the additive in rapid crystallization under experimental conditions.

Chlorophylls and their derivatives are thermally stable in the range from 20 to 200 °C, and thermal decomposition begins no earlier than at 300 °C [61]. According to this, we can evaluate the effect of additives on the formation of the supramolecular structure of the PHB, focusing on the thermophysical properties of the polymer. Thus, the first heating allows us to assess the state of the polymer in the form of fibers, the supramolecular structure of which is largely determined by the peculiarities of the electroforming process [62]. The second heating allows us to see the thermophysical behavior of the native state of PHB formed under the influence of chlorophyll derivatives.

It should be mentioned that the melting temperature of PHB changes slightly, and therefore the size of the crystallites remains quite close, because the larger crystallites melt at higher temperatures and smaller ones at lower temperatures [63]. It is important to note that the introduction of all additives reduced the enthalpy of melting by 8–17% at the first melting and by 12–19% at the second melting. These data indicate the plasticizing effect of additives. Moreover, it is important to note that the type of the selected radical, as can be seen from the results, influenced the structure of the PHB in its own way. This is especially evident in the form of peaks during the second heating. It can be seen that when all additives are introduced, a low-temperature shoulder appears in the range of 155–165 °C. This shoulder indicates the formation of defective crystallites or a fine crystalline fraction during the formation of fibers. And consequently, at the stage of forming of the polymer melt, additives can prevent the slow crystallization of a more regular fraction of PHB, acting as crystallization aggregates.

This is probably due to the intermolecular interaction of PHB and chlorophyll derivatives. The most likely are hydrogen bonding between an ethereal bond and R1 radical and ionic attraction between carbonyl- and nitrogen-containing groups [64].

The most notable for the identification and evaluation of PHB are the following peaks: C=O carbonyl at 1720 cm^−1^, -C-O-C- ester linkage at 1150–1300 cm^−1^, -C-H bonds in methyl radical at 2900–3100 cm^−1^, and -CH_3_ at 1380 cm^−1^ [65]. The region 400–1300 cm^−1^ is responsible for a complicated series of absorptions due to all manner of bending vibrations of PHB [66]. It is important to mention C-C vibrations of the carbon skeleton at 980 cm^−1^, O-C-C stretching at 1053 cm^−1^ and 1130 cm^−1^, and C-O stretching at 1225 cm^−1^ and 1270 cm^−1^ [67].

Upon closer examination of this area (Figure 4b), it can be clearly seen that the additive does not change the chemical structure of the PHB. The most characteristic peaks for chlorophyll derivatives can be considered peaks of the aldehyde group at 2850 cm^−1^ and carbonyl group at 1720 cm^−1^ [68]. These peaks are largely overlapped by the peaks of the PHB, as can be seen from Figure 4a. In addition, as can be seen from Figure 4b, the peaks that are responsible for C-N stretching vibrations at 1380 cm^−1^ and C-O stretching vibrations at 1051 cm^−1^ are also superimposed on the peaks of PHB, and even their relative intensity does not change [69].

The observed effect is due to a very low concentration of additives (0.03%) in the material and a low probability of its localization on the fiber surface.

In works by Luo et al. [67], Kovalcik et al. [70], and Xu et al. [71] the authors proposed to evaluate the state of the crystalline phase of PHB with the ratio of the peak intensities of 1225 cm^−1^ and 1453 cm^−1^, which allows us to characterize changes in the size of the crystalline fraction of the PHB. The following ratios were obtained: 3.63 for PHB; 3.64 for PHB/mC_4_; 3.22 for PHB/mC_3_OH; 3.59 for PHB/mC_2_NH_2_; 3.88 for PHB/mC_2_N+; and 3.13 for PHB/mC_2_N. These ratios are very close values and indicate weak changes in the structure of the crystalline phase of PHB. However, this method should be subjected to some criticism, since the signal is taken from the surface (depth of penetration of the IR beam into the sample—approximately 2 μm, which is comparable to the average fiber diameter) and does not give a complete picture of the structural organization in the mass of fibers; in addition, ATR-FTIR is not a quantitative technique able to show a full picture of crystalline structure of PHB in all layers of the nonwoven materials.

The chemical structure of PHB contains a hydrophobic methyl group and hydrophilic ester groups, but in general, PHB is a hydrophobic polymer [72]. The control of its hydrophilicity is an important task, the solution to which can greatly expand the areas of biomedical application of this polymer [73]. In the works of Russie et al., the authors were able to obtain hydrophobic materials based on chlorophyll derivatives [74]. The greatest increase in hydrophilicity can be seen for mC_3_OH, and to a less extent for mC_2_N, which may be due to the appearance of open pores on the surface of fibers that contribute to wettability. The mC_2_NH_2_ additive has even less effect. An increase in the hydrophilicity of the material can occur due to the presence of a polar amino group, which is able to form hydrogen bonds with water molecules. In other cases, we see a slight increase in hydrophobicity, which is inherent in chlorophyll derivatives.

Many tetrapyrroles have notable antimicrobial characteristics [75,76,77]. Chlorophyll derivatives are considered good antimicrobial agents in the case of photodynamic therapy. Due to singlet oxygen, the antimicrobial effect of chlorophyll derivatives has been shown in relation to *Staphylococcus aureus*, *Escherichia coli*, *Candida albicans*, and *Artemia salina* in works by Gerola et al., Indrawati et al., and Suvorov et al. [74,78,79]. However, of interest is the ability of tetrapyrroles to exhibit antimicrobial properties in the absence of excitation, but for the time being in the material. To answer this question, samples of PHB/chlorophyll derivatives electrospun materials were placed in medium with *E. Coli*.

Thus, it was observed that the electrospun materials themselves do not have antimicrobial properties, but they suppress the activity of *E. coli* directly under the material layer. The lysis zone does not go beyond the boundaries of the material; moreover, it practically does not differ from the one obtained for pure PHB. 

This is due the absence of photo excitation. This observation can be an important aspect in the planning of safe carriers of porphyrins.

It is important to note that materials based on PHB are characterized by low strength and high brittleness. Often, biomimetic medical materials are not required to withstand high loads, so this is not a problem. In many respects, high brittleness is a consequence of the semi-crystalline nature of the polymer, but also, due to the low glass transition temperature, the amorphous phase is in a glazed state and is quite fragile. The glass transition temperature of PHB is 4–7 °C [80].

A number of scientists note that various additives are able to affect the glass transition temperature of PHB, which indicates changes in the state of the amorphous phase and, as a consequence, the mechanical properties [81,82].

So, with the introduction of additives, we see the appearance of a peak in the range 47–50 °C (Figure 5 1 heating), as in works by Ioradanskii et al. [83]. These peaks may indicate a shift in the glass transition temperature, which means that the additive is localized in the amorphous phase. Thus, at room temperature, the amorphous phase of PHB with chlorophyll derivatives is in an elastic rubber state. However, this did not allow us to increase or improve the mechanical properties of nonwovens based on PHB. The tensile strength of the materials decreased by 11%. In general, materials for PT, as a rule, do not have high strength requirements; it is only necessary to overcome the minimum required to ensure operation, which is 1–1.5 MPa. The decrease in strength despite the improvement in the morphology of the material should be attributed to the result of changes in the supramolecular structure.

Elongation decreased by 60% in the case of PHB/mC_4_ and by 80% in the case of PHB/mC_2_N+, despite the fact that the number of defects decreased, and the state of the amorphous phase changed. Despite the reduction of the morphological defects, the introduction of the additives initiates rapid destruction of the material in the case of an applied loads. This may be a consequence of the noticeable effect of the photosensitizers on the state of the amorphous phase, which was noted earlier. In addition, as can be seen from the results of the DSC (Figure 5), the crystalline phase is in a less regular state, and a significant part of the crystallites is represented by a defective fraction, which does not have time to crystallize during the second heating and melts earlier in the range of 155–165 °C.

## 5. Conclusions

This investigation characterizes the new system of PHB/chlorophyll derivatives electrospun materials. The capabilities of this system show the possibility of developing new flexible carriers with low concentrations of chlorophyll derivatives. These new materials are an example of improved flexible carriers of drugs. 

The investigation was successful because it provided an opportunity to solve the problem of creating biocompatible flexible carriers capable of delivering small concentrations of dosage forms to the target.

In the study, new materials based on the biopolymer poly-3-hydroxybutyrate (PHB) modified with chlorophyll derivatives were proposed. These electrospun materials possess biomimetic characteristics. All five examined chlorophyll derivatives enabled the production of nonwoven materials through the electrospinning method. However, mC_2_N had a negative impact on the mechanical properties, making this additive unsuitable for any application.

Summing up the resulting impact of the investigated additives on polymeric matrixes of PHB, the following should be mentioned:

- mC_4_, mC_3_OH, mC_2_NH_2_, and mC_2_N+ enable the decreasing of the average diameters of the electrospun materials by 25–40% and increase air permeability by 600–1040%. This can be an excellent achievement when planning highly porous permeable materials with membrane properties.
- The introduction of additives clearly accelerated the crystallization of the polymer, reducing the degree of crystallinity of PHB by 6–10%, and also had an effect on the state of the amorphous phase. Reducing the crystalline phase of natural polyesters such as PHB allows for the control of the rate of their bioresorption and biodegradation, which can be a valuable advantage when planning deliveries with a controlled life span.
- mC_3_OH enabled the significant hydrophobization of the surfaces of the nonwoven material. As an addition to the proportion of open pores, this aspect is important for planning deliveries to areas where it is important to increase cell proliferation by attaching them to a more hydrophilic surface.

All other additives led to a reduction in the crystallinity of PHB while preserving the highly developed material structure, which led to a great impact on the air permeability. Additives mC_4_ and mC_2_N+ resulted in a decrease in structural defects, with mC_4_ producing the thickest fibers and mC_2_N+ achieving the lowest bulk density of the material. These additives can be employed to control the hydrophilicity and hydrophobicity of the produced material. 

We have researched the most important characteristics of required materials for solving the problem of cancer therapy, such as highly developed structure, flexibility of the system, possibility of effective modification, and biocompatibility. Also, the obtained materials based on PHB/mC_4_ and PHB/mC_2_N+, due to high permeability, lower crystallinity, and highly developed hydrophilic surface, could be used in creating grafts and implants because these characteristics are important for better cell growth. So, further studies of these materials should be considered promising. 

At the next stage of the study, we propose to consider the combinations of PHB/chlorophyll derivatives electrospun systems with PHB/mC_4_ and PHB/mC_2_N+ with satisfactory mechanical properties high enough to create delivery systems with controlled morphologies and to study their activation behavior, drug release profiles, and cytotoxicity on tumor cells. This will enable the optimization of parameters for the efficient delivery of photosensitizers in biological systems. Additionally, studying cytotoxicity will further confirm the potential of these materials for application in oncology and photodynamic therapy. Attention to the details of activation behavior and release profiles will further enhance the understanding of the influence of structure on the biological activity of these materials, crucial for their successful medical application.

## Figures and Tables

**Table 1:** Features of the ES process of PHB/chlorophyll derivatives electrospun materials.

Sample	Voltage, Kv	Viscosity, Pa s	Flow Rate, mL/min
PHB	18	1.0	6.6
PHB/mC_2_NH_2_	17	1.2	6.6
PHB/mC_2_N	19	1.2	7.2
PHB/mC_2_N+	17	1.2	4.8
PHB/mC_4_	17	1.2	6.4
PHB/mC_3_OH	17	1.2	6.6

**Table 2:** Morphology of PHB/chlorophyll derivatives electrospun materials.

Sample	Average Diameter, µm Δ ±0.04 µm	Bulk Density, g/cm^3^ Δ ±0.01 g/cm^3^	Air Permeability, mL Δ ±0.2 ml	Time by Gurley Method, s Δ ±0.6 s
PHB	3.5	0.30	0.38	50.0
PHB/mC_2_NH_2_	2.24	0.18	3.75	26.7
PHB/mC_2_N	4.86	-	-	-
PHB/mC_2_N+	2.27	0.13	2.78	36.0
PHB/mC_4_	2.63	0.16	2.35	42.6
PHB/mC_3_OH	2.08	0.16	4.35	23.0

**Table 3:** Thermal properties of PHB/chlorophyll derivatives, where χ—crystallinity degree Δ ±2.5%, ΔH—melting enthalpy Δ ±2.5%, and T_m_—melting temperature Δ ±2%.

Sample	First Heating Run			Second Heating Run		
	T_m_, °C	ΔH, J/g	χ, %	T_m_, °C	ΔH, J/g	χ, %
PHB	175	93.1	63.8	170	90.8	62.2
PHB/mC_2_NH_2_	176	83.3	57.1	174	78.7	53.9
PHB/mC_2_N	176	85.8	58.8	173	81.2	55.6
PHB/mC_2_N+	177	80.6	55.2	173	79.8	54.7
PHB/mC_4_	176	82.0	56.2	174	78.9	54.0
PHB/mC_3_OH	175	77.4	53.0	171	74.9	51.3

**Table 4:** Mechanical properties of PHB/chlorophyll derivatives electrospun materials.

Sample	Tensile Strength, MPa Δ ±0.02 MPa	Elongation at Break, % Δ ±0.2%
PHB	1.7	3.6
PHB/mC_2_NH_2_	-	-
PHB/mC_2_N	-	-
PHB/mC_2_N+	1.5	1.3
PHB/mC_4_	1.5	0.6
PHB/mC_3_OH	-	-
